# Impaired influenza A virus replication by the host restriction factor SAMHD1 which inhibited by PA-mediated dephosphorylation of the host transcription factor IRF3

**DOI:** 10.1186/s12985-024-02295-0

**Published:** 2024-01-29

**Authors:** Zhilei Zhao, Shuyi Han, Qingxun Zhang, Ye Wang, Kening Yue, Salbia Abbas, Hongxuan He

**Affiliations:** 1 National Research Center for Wildlife-Borne Diseases, Institute of Zoology, Chinese Academy of Sciences, Beijing, 100101 China; 2 University of Chinese Academy of Sciences, Beijing, 100101 China; 3 Beijing Milu Ecological Research Center, Beijing, 100076 China

**Keywords:** SAMHD1, H5N1, PA, Host restriction factor, Influenza virus

## Abstract

**Background:**

Influenza A virus (IAV) can cause severe and life-threatening illness in humans and animals. Therefore, it is important to search for host antiviral proteins and elucidate their antiviral mechanisms for the development of potential treatments. As a part of human innate immunity, host restriction factors can inhibit the replication of viruses, among which SAM and HD domain containing deoxynucleoside triphosphate triphosphohydrolase 1 (SAMHD1) can restrict the replication of viruses, such as HIV and enterovirus EV71. Viruses also developed countermeasures in the arms race with their hosts. There are few reports about whether SAMHD1 has a restriction effect on IAV.

**Methods:**

To investigate the impact of IAV infection on SAMHD1 expression in A549 cells, we infected A549 cells with a varying multiplicity of infection (MOI) of IAV and collected cell samples at different time points for WB and RT-qPCR analysis to detect viral protein and SAMHD1 levels. The virus replication level in the cell culture supernatant was determined using TCID50 assay. Luciferase assay was used to reveal that H5N1 virus polymerase acidic protein (PA) affected the activity of the SAMHD1 promoter. To assess the antiviral capacity of SAMHD1, we generated a knockdown and overexpressed cell line for detecting H5N1 replication.

**Results:**

In this study, we observed that SAMHD1 can restrict the intracellular replication of H5N1 and that the H5N1 viral protein PA can downregulate the expression of SAMHD1 by affecting SAMHD1 transcriptional promoter activity. We also found that SAMHD1's ability to restrict H5N1 is related to phosphorylation at 592-tyrosine.

**Conclusions:**

In conclusion, we found that SAMHD1 may affect the replication of IAVs as a host restriction factor and be countered by PA. Furthermore, SAMHD1 may be a potential target for developing antiviral drugs.

## Background

Influenza viruses are divided into four subtypes: A, B, C and D. Influenza virus subtypes A, B, and C are capable of infecting humans, and the virus causing many times pandemics is IAV. It has caused many major pandemics, such as the 1918 “Spanish influenza” (H1N1) [1], 1957 “Asian influenza” (H2N2) [2–4], 1968 “Hong Kong influenza” (H3N2) [5, 6] and 2009 H1N1 influenza [7]. Together, it killed an estimated 50 million people worldwide [8, 9]. Currently, IAV still causes 10% of human infections and approximately half a million deaths each year [10].

Hemagglutinin (HA) and neuraminidase (NA) are the main viral proteins that cause antigenic responses on the virus surface [11]. IAV is subtyped according to the antigenicity of the surface glycoproteins into 16 HA and 9 NA subtypes. It can form a variety of subtypes through antigenic drift and antigenic shift, among which H1N1 and H3N2 are circulating in the population, causing widespread transmission. Other viruses such as H5N1, H5N6, H6N1, H7N2, H7N3, H7N4, H7N7, H7N9, H9N2, H10N7, and H10N8 pose a potential risk to humans [12]. To date, all IAVs that cause pandemics are produced by genetic reassortment [13–15]. Among avian influenza virus (AIVs), highly pathogenic avian influenza (HPAI) H5N1 has the most serious impact on human health [16]. In 1997, H5N1 virus was first reported in Hong Kong, China, and caused acute pneumonia and death [17, 18]. In 2003, H5N1 reemerged in Asia, and by 2019, 861 patients had been diagnosed, of whom 455 patients had died [19]. In 2020, virus 2.3.4.4b, a branch of HPAI H5N1, was reported to the World Health Organization (WHO). The virus originated from the previously circulating H5Nx virus and was a recombination of the H5N8 with the H1N1 and H3N8 subtypes of AIV [20]. This virus has resulted in many deaths in wild birds and poultry, which means that virus 2.3.4.4b is now widespread in the wild [21]. This indicates that H5N1 still poses a potential pandemic risk to animals and humans.

After IAV enters cells, host cells recognize the virus through pattern recognition receptors (PRRs), which induce the secretion of cytokines such as type I interferon (IFN-I). IFN-I binds to IFN receptors to activate downstream signaling pathways. It leads to the transcription of hundreds of interferon stimulator genes (ISGs), which puts cells into an “antiviral state” and suppresses the replication and spread of viruses [22]. In contrast, IAV have also evolved several strategies to hinder IFN expression, thus successfully proliferating in cells [23]. During IAV replication in cells, nonstructural protein 1(NS1) and PA can inhibit the IFN signaling pathway, thus bypassing the antiviral activity of the host immune system, which is critical for IAV infection [24–27]. PA participates in the life cycle of IAV and is closely related to virulence. Its amino acid mutation can affect the virulence and pathogenicity of the virus [28]. Some cellular proteins can interact with PA and are required for viral replication, such as C14orf166 (chromosome 14 open reading frame 166, CLE) [29] and minichromosome maintenance (MCM) complex [30]. However, some host proteins interacting with PA proteins to restrict virus replication have also been identified, such as HCLS1-associated protein X-1(HAX1) [31].

Many host restriction factors can limit the replication of viruses, and some host restriction factors also belong to ISGs, such as ISG15 [32]. The expression of SAMHD1 in cells is also upregulated by IFN [33]. miR-181a, miR-155 and miR-30a downregulate SAMHD1 expression by binding to the 3’UTR [33, 34]. In contrast, SAMHD1 participates in the IFN pathway as a negative regulator [35]. SAMHD1 plays a key role in regulating the cell cycle, innate immune responses, and DNA damage repair. Mutation of the human SAMHD1 protein is associated with the autoimmune disease Aicardi-Goutières syndrome (AGS). When SAMHD1 has a loss-of-function mutation, the patient's main clinical symptom is increased production of IFN-I throughout the whole body [36]. SAMHD1 was identified as an inhibitor of HIV-1 in 2011 [37–39]. It is a 72 kDa protein that can be divided into four functional regions, namely, the nuclear localization sequence (NLS), the SAM region associated with tetramer formation, the HD region associated with enzyme activity, and the VPX binding region [40, 41]. SAMHD1 inhibits early-phase HIV-1 replication in dendritic and other myeloid cells by preventing viral cDNA synthesis via its negative regulatory effect on intercellular dNTP pools [42, 43]. Meanwhile, some reports suggest that the nuclease activity of SAMHD1 contributes to its antiviral activity by directly degrading HIV-1 RNA [44–46]. Furthermore, SAMHD1 has also been reported to regulate HIV-1 latency by suppressing HIV-1 long terminal repeats (LTR) activity [47]. In addition, there is growing evidence that SAMHD1 inhibits various viruses, including retroviruses and DNA viruses, by depleting the dNTP pool in cells [45, 48–52], making it insufficient to support viral DNA synthesis [53–56].

Our previous studies have shown that SAMHD1 effectively inhibits the replication of RNA viruses such as Enterovirus 71 (EV71), while TRIM21, an E3 ligase, and IFN inducer, interacts specifically with and degrades SAMHD1 via the proteasome pathway [57]. Although SAMHD1 has a restriction effect on HIV and EV71, its restriction effect on IAV has not been reported, and whether IAV can overcome SAMHD1's antiviral effect to infect cells remains to be investigated. In this study, we describe the restriction effect of SAMHD1 on H5N1 and the mechanism by which H5N1 downregulates SAMHD1 via PA. These findings extend the study of the antiviral mechanism of SAMHD1 and contribute to the development of new antiviral strategies.

## Materials and methods

### Plasmid construction

PLKO.1-shSAMHD1 was kindly provided by Professor Wenyan Zhang (Jilin University) [57]. SAMHD1 followed by a his/myc tag was inserted into BamHI/XhoI of pCDNA3.1. Whole mRNAs including cells mRNA and viral mRNA were extracted with Trizol (Invitrogen, Carlsbad, CA, USA) and reverse transcripted (Invitrogen) according to the manufacturer’s instructions. Interferon regulatory Factor 3(IRF3) and viral PA followed by a flag tag were amplified from viral mRNAs and inserted into BamHI/XhoI of pCDNA3.1. Mutations of SAMHD1-mychis were constructed on SAMHD1-mychis with primers listed in Table 1.

**Table 1:** Primers used in this study

Primer name	Sequence
SAMHD1-BAMH1-F	CGGGATCCACCATGCAGCGAGCCGATTCC
SAMHD1-XHO1-R	CGCTCGAGCATTGGGTCATCTTTAAAAAGCTGGACT
SAMHD1-XHOI-FLAG-F	CCCTCGAGACCATGGACTACAAGGACGACGATGACAAGCAGCGAGCCGATTCC
SAMHD1-BAMHI-R	CGGGATCCTCACATTGGGTCATCTTTAAAAAGCTGGA
SAM-T592A-F	AGCCCCACTCATAGCACCTCAAAAAAAGG
SAM-T592D-F	AGCCCCACTCATAGACCCTCAAAAAAAGGAAT
SAM-592-R	CTATGAGTGGGGCTATAACATCG
IRF3-ECORI-F	CGGAATTCATGGGAACCCCAAAGCC
IRF3-XHOI-R	CGCTCGAGTTGGTTGAGGTGGTGGG
SAMHD1pro-nheI-F	CGGCTAGCACTTAATTCATTTAGTATT
SAMHD1pro-xhoI-R	CCCTCGAGGGCTACACCTGGCGTCCGG
SAMHD1-RT-F	GGTCCGGAGCAGGTGTG
SAMHD1-RT-R	CAAAACGAGACTCATCAAGAC
GAPDH-RT-F	CCCATCACCATCTTCCAGG
GAPDH-RT-R	TTCTCCATGGTGGTGAAGAC
NP-RT-F	AGGCACCAAACGGTCTTACG
NP-RT-R	TTCCGACGGATGCTCTGATT

### Stable silenced and overexpressed cell lines

For stable silenced cell lines, HEK293T cells were cotransfected with sh-SAMHD1-PLKO.1 or PLKO.1 plus RRE, REV, and VSV-G expression vectors by using Lipofectamine 3000 (Invitrogen). For stable overexpressed cell lines, 293T cells were cotransfected with SAMHD1-PLVX or PLVX plus RRE, REV, and VSV-G expression vectors by using Lipofectamine 3000 (Invitrogen). At 48 h after transfection, supernatants containing packaged lentivirus were harvested and used to infect A549 cells for 48 h. Puromycin (1.5 μg/ml for A549, Sigma, St. Louis, MO, USA) was then added to the culture to screen for stable cell lines.

### Cell culture and viruses

Human lung alveolar epithelial cells A549, HEK293T cells and MDCK cells were cultured as monolayers in Dulbecco’s modified Eagle’s medium (DMEM) (HyClone, Logan, UT, USA) supplemented with 10% fetal calf serum (FCS, GIBCO BRL, Grand Island, NY, USA) and 1% penicillin‒streptomycin and maintained at 37 °C in a humidified atmosphere with 5% CO_2_. Influenza virus A/environment/Qinghai/1/2008(H5N1) and H1N1 virus A/Beijing/05/2009 were grown in 8-day-old embryonated chicken eggs and titrated on MDCK cells.

### Transfection and infection

DNA transfections were conducted by Lipofectamine 3000 (Life Technologies) according to the manufacturer's instructions. For viral infection, briefly, A549 cells and 293T cells were grown to 70–80% confluence in a 12-well plate, washed twice with phosphate-buffered saline (PBS) and incubated with IAVs at 37 °C for 1 h at the described MOI. The DMEM supplemented for virus infection contained 2% FBS and 2.5% bovine serum albumin (BSA). During adsorption, the plate was gently agitated at 20-min intervals. Following adsorption, the virus-containing medium was replaced with fresh medium containing 2% FCS, followed by incubation at 37 °C in 5% CO_2_ for the indicated time points.

### TCID50

First, 1.5 × 10^4^ MDCK cells were seeded in a 96-well plate one day before infection. Twenty-four hours later, the virus was diluted to different dilutions with fresh serum-free DMEM containing 2.5% BSA. Then, MDCK cells were infected with the diluted virus for 1 h and gently agitated at 20-min intervals. Before and after infection, the inoculum was removed and washed once with PBS. The positive cytopathic effects correlating with the amount of virus were documented and used for calculating the virus titer according to Reed and Muench’s method. Each experimental condition was tested in triplicate.

### Immunoblotting (IB) and antibodies

Transfected or infected cells were harvested and boiled in 1X loading buffer (0.08 M Tris, pH 6.8, with 2.0% SDS, 10% glycerol, 0.1 M dithiothreitol and 0.2% bromophenol blue) followed by separation on a 12% polyacrylamide gel. Proteins were transferred onto a nitrocellulose (NC) membrane for Western blot analysis (Pall, catalog no. 66458). The membranes were incubated with primary antibodies, followed by a corresponding horseradish peroxidase (HRP)-conjugated secondary antibody (Jackson Immunoresearch, West Grove, PA, USA) diluted 1:10,000. Proteins incubated with HRP-conjugated secondary antibody were visualized using the ultra-sensitive ECL chemiluminescence detection kit (Meilunbio, catalog no. MA0186).

### RNA extraction and RT‒qPCR

Total cell RNA was extracted from infected A549 or 293T cells with Trizol reagent (Invitrogen). Two micrograms of RNA were reverse transcribed into cDNA using the GoScript™ Reverse Transcriptase System (Promega, A5004). RT‒qPCR was performed on an ABI 7500 Fast Real-Time PCR system (Applied Biosystems, Carlsbad, CA) with the UltraSYBR Mixture (Cwbio, CW2601M) in a total volume of 20 μl per sample, containing 10 μl of 2 × mix, 1 μl of 5 μmol/L of each primer and 2 µg of cDNA template. The primers used in RT‒qPCR is presented in Table 1.

### Luciferase reporter assay

HEK293T cells were plated in 12-well culture plates at 70–80% confluence and transfected with the indicated plasmids along with 0.02 µg pRL-TK as an internal reference control by Lipofectamine 3000 (Life Technologies). Twenty-four hours after transfection, the cells were harvested and subjected to a luciferase assay.

### Statistical analysis

The detailed statistical analysis is described in the figure legends. All data are expressed as the mean ± standard deviation (SD). Statistical comparisons between the two groups were made using Student’s t-test. Significant differences are indicated in the figures as follows: *p < 0.05, **p < 0.01 and ***p < 0.001. *p* values of less than 0.05 are considered to represent a statistically significant difference, ns stands for no significance.

## Results

### SAMHD1 expression was downregulated by H5N1 virus infection

**Fig. 1:**
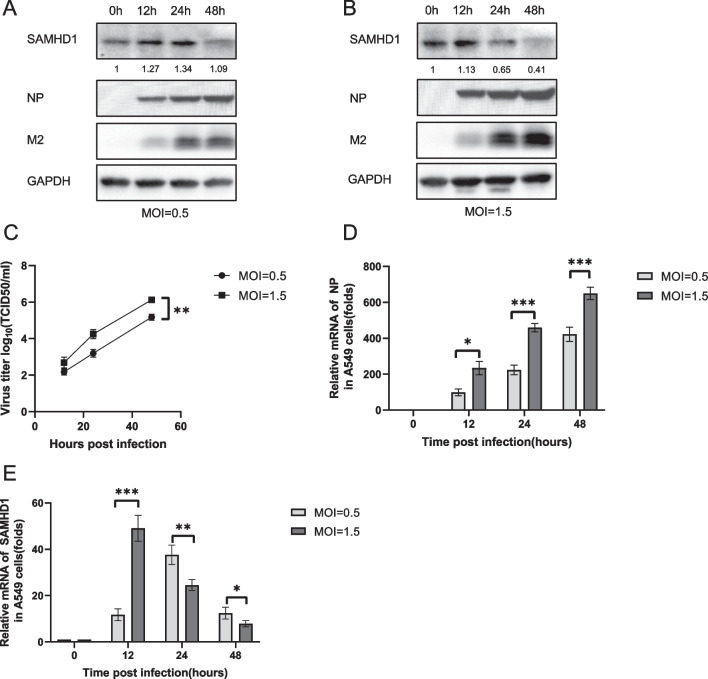
Changes in SAMHD1 protein levels and RNA levels in A549 cells infected with H5N1. A549 cells were infected with H5N1 at MOIs of 0.5 (**A**) and 1.5 (**B**), respectively. The cells and culture supernatants were harvested at the indicated time points. Immunoblotting (IB) analysis of H5N1 NP, M2, and SAMHD1 in cells was performed, with GAPDH as a loading control (**A**, **B**). The expression levels of SAMHD1 protein were quantified with ImageJ software to calculate the values relative to that for GAPDH. The infectious viral loads in the cell supernatants were determined by TCID50 analysis using 96-well plates (**C**). H5N1 NP (**D**) and SAMHD1 (**E**) RNA levels in cell lysates were detected by RT‒qPCR and normalized to GAPDH, and the RNA level in A549 cells at 0 h post infection was set as 1. Data are the mean ± SD of three independent experiments. The results are representative of those from three independent repeats (mean ± SD, *p < 0.05, **p < 0.01, paired *t* test)

**Fig. 2:**
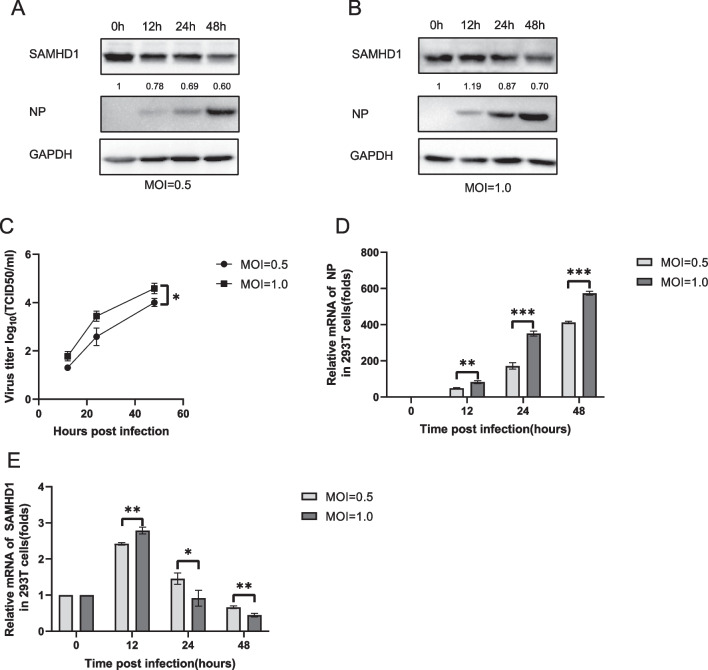
Changes in SAMHD1 protein levels and RNA levels in 293T cells infected with H5N1. 293T cells were infected with H5N1 at MOIs of 0.5 (**A**) and 1.0 (**B**). The cells and culture supernatants were harvested at the indicated time points. Immunoblotting (IB) analysis of H5N1 NP and SAMHD1 in cells was performed, with GAPDH as a loading control (**A**, **B**). The expression levels of SAMHD1 protein were quantified as described before. The infectious viral loads in the cell supernatants were determined by TCID50 analysis using 96-well plates (**C**). H5N1 NP (**D**) and SAMHD1 (**E**) RNA levels in cell lysates were detected by RT‒qPCR and normalized to GAPDH, and the RNA level in A549 cells at 0 h post infection was set as 1. Data are the mean ± SD of three independent experiments. The results are representative of those from three independent repeats (mean ± SD, *p < 0.05, **p < 0.01, ***p < 0.001, paired t test)

IAV, like other viruses, needs to overcome host immunity to replicate in cells. It can hinder interferon production in a variety of ways. To investigate the interaction between IAV and SAMHD1, we infected A549 cells with H5N1 at a MOI of 0.5 and 1.5. The cells and culture supernatants were collected at different time points. The protein and RNA levels of the viral protein nucleoprotein (NP) increased significantly with time (Fig. 1A, B, D). The H5N1 titer of the cell culture supernatant also increased with time (Fig. 1C). The protein level of SAMHD1 increased slightly at 24 and 12 h after infection and then decreased (Fig. 1A, B). The RNA level of SAMHD1 in A549 cells also showed a trend of first increasing and then decreasing after virus infection (Fig. 1E). Similar to the SAMHD1 protein level shown in the WB results, the decrease in SAMHD1 RNA was more significant at an MOI of 1.5 than at an MOI of 0.5 (Fig. 1E). In conclusion, the results suggest that H5N1 may downregulate SAMHD1 expression by affecting transcription stage. In addition to A549 cells, we also infected 293T cells with H5N1 and monitored the viral replication status and the amount of SAMHD1 at various time points. The results showed that the findings in 293T cells were similar to those in A549 (Fig. 2). However, although SAMHD1 in 293T cells was upregulated and then decreased after infection, the change was less significant than that in A549 cells (Fig. 2E).

### The downregulation of SAMHD1 expression was correlated with the MOI of H5N1

**Fig. 3:**
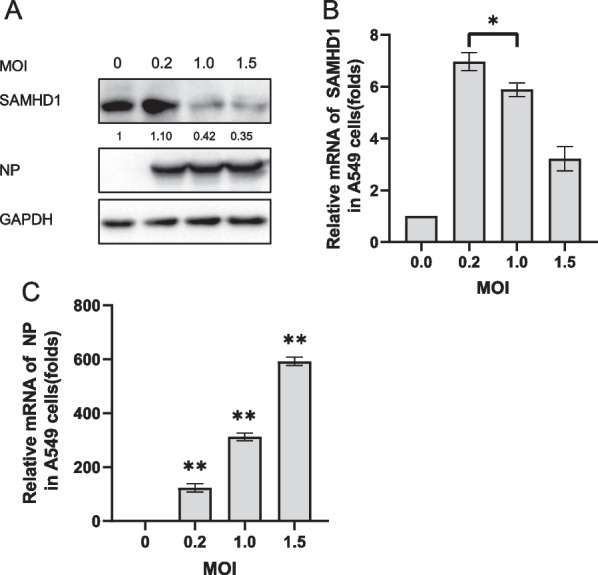
Effect of H5N1 infection dose on SAMHD1 protein levels and RNA levels in A549 cells. A549 cells were infected with H5N1 at an MOI of 0.2, 1.0, or 1.5 (**A**). The cells were harvested at 48 h post infection. Immunoblotting (IB) analysis of H5N1 NP and SAMHD1 in cells was performed, with GAPDH as a loading control (**A**). The expression levels of SAMHD1 protein were quantified as described before. H5N1 NP (**B**) and SAMHD1 (**C**) RNA levels in cell lysates were detected by RT‒qPCR and normalized to GAPDH, and the RNA level in A549 cells at 0 h post infection was set as 1. Data are the mean ± SD of three independent experiments. The results are representative of those from three independent repeats (mean ± SD, * *p* < 0.05, ** *p* < 0.01, ***p < 0.001, paired t test)

To further confirm the above results and demonstrate the effects of different H5N1 MOIs on A549 cells, we infected A549 cells with H5N1 at MOIs of 0.2, 1.0, and 1.5 and analyzed the expression of SAMHD1 and NP at 48 h post-infection. We observed that the viral protein NP increased with the infection titer (Fig. 3A, C). The expression of SAMHD1 protein was significantly downregulated with increasing infection dose (Fig. 3A), suggesting that H5N1 virus infection can downregulate SAMHD1 expression in A549 cells, and as the infection titer increases, the amount of SAMHD1 protein in the cells decreases. However, after infecting cells with H5N1 at MOIs of 1.0 and 1.5, the amount of SAMHD1 mRNA did not decrease as significantly as the protein level (Fig. 3A, B), and the RNA levels of SAMHD1 in samples infected with H5N1 at MOIs of 1.0 and 1.5 were still higher than those uninfected after 48 h of infection (Fig. 3B), consistent with the results at the 48-h time point in Fig. 1E. This could be due to the protein expression of SAMHD1 being subject to posttranscriptional regulation. The results showed that H5N1 downregulates SAMHD1 at the protein and mRNA levels. Its downregulation ability was positively related to the MOI of the infected virus.

### SAMHD1 expression was downregulated by H1N1 virus infection

**Fig. 4:**
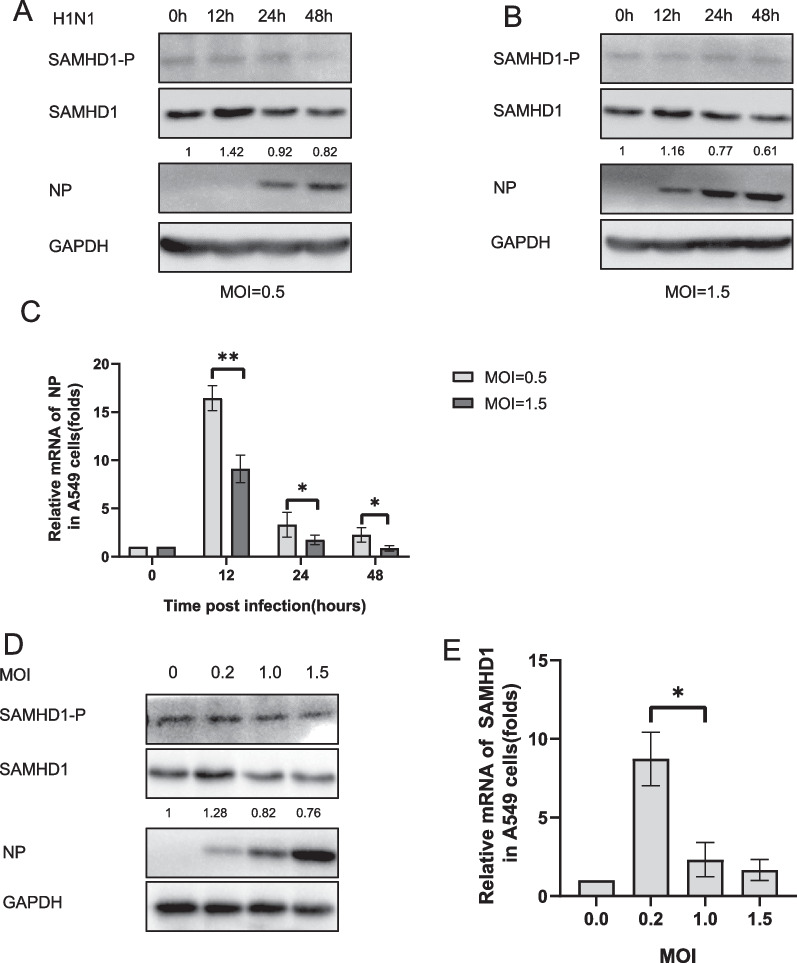
Changes in SAMHD1 protein and RNA levels in A549 cells infected with H1N1. A549 cells were infected with H1N1 at MOIs of 0.5 (**A**) and 1.5 (**B**), respectively. The cells were harvested at the indicated time points. IB analysis of H1N1 NP, SAMHD1 and phosphorylated SAMHD1(SAMHD1-P) in cells was performed, with GAPDH as a loading control. The expression levels of SAMHD1 protein were quantified as described before (**A**, **B**). A549 cells were infected with H1N1 at an MOI of 0.2, 1.0, or 1.5 (**D**). The cells were harvested at 48 h post infection. IB analysis of H1N1 NP and SAMHD1 in cells was performed, with GAPDH as a loading control. The expression levels of SAMHD1 protein were quantified as described before (**D**). SAMHD1 RNA levels in cell lysates were detected by RT‒qPCR and normalized to GAPDH, and the RNA level in A549 cells at 0 h post infection was set as 1 (**C**, **E**). Data are the mean ± SD of three independent experiments. The results are representative of those from three independent repeats (mean ± SD, *p < 0.05, **p < 0.01, paired t test)

**Fig. 5:**
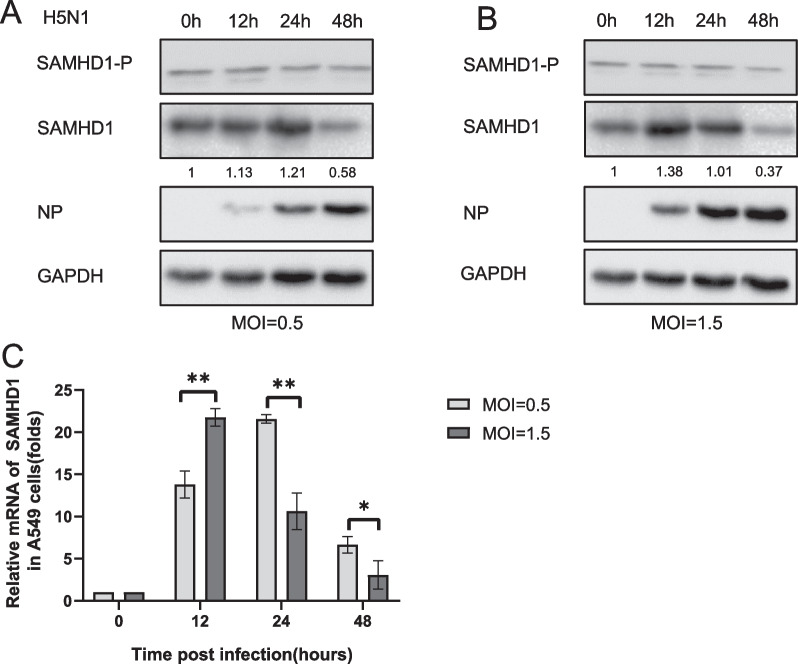
Effect of H5N1 on phosphorylation of SAMHD1. A549 cells were infected with H5N1 at MOIs of 0.5 (**A**) and 1.5 (**B**), respectively. The cells were harvested at the indicated time points.IB analysis of H5N1 NP, SAMHD1and SAMHD1-P in cells was performed, with GAPDH as a loading control (**A**, **B**). The expression levels of SAMHD1 protein were quantified with ImageJ software to calculate the values relative to that for GAPDH. SAMHD1 RNA levels in cell lysates were detected by RT‒qPCR and normalized to GAPDH, and the RNA level in A549 cells at 0 h post infection was set as 1 (**C**). Data are the mean ± SD of three independent experiments. The results are representative of those from three independent repeats (mean ± SD, *p < 0.05, **p < 0.01, paired t test)

Through the above results, we found that H5N1 can down-regulate SAMHD1 expression and is correlated with the amount of virus. To see if other IAV have similar capabilities, we conducted a similar experiment with H1N1. we infected A549 cells with H1N1 at several MOIs and analyzed the expression of SAMHD1 (Fig. 4). At the same time, since the phosphorylation of SAMHD1 may affect its antiviral ability[58, 59], we also examined the effect of influenza virus infection on SAMHD1 phosphorylation (Figs. 4, 5). By examining the changes of SAMHD1 after H5N1 and H1N1 different MOI infection, we found that the down-regulation effect of IAV on SAMHD1 is broad spectrum. Both H5N1 and H1N1 can downregulate SAMHD1 expression. Phosphorylation levels were not significantly associated with IAV infection Fig 4 (A, B, D) , (Fig. 5A). The ability of H5N1 to down-regulate SAMHD1 appears to be more significant than H1N1. This may be related to the pathogenicity of the virus. We take H5N1 as the main research content in next study.

### H5N1 downregulates SAMHD1 by affecting its promoter activity rather than degrading the protein

**Fig. 6:**
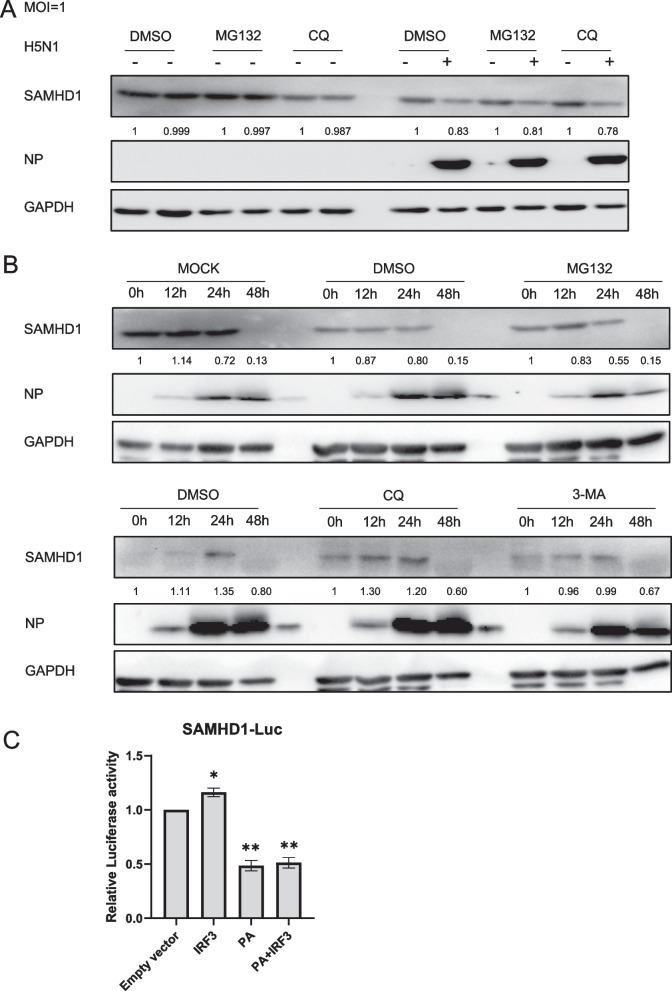
Mechanism of H5N1 downregulation of SAMHD1 protein and RNA levels. A549 cells were infected with H5N1 at an MOI of 1.0 (**A**, **B**). A549 cells were treated with 0.1% dimethyl sulfoxide (DMSO), 10 μM MG132, 50 μM CQ and 160 μM 3-MA for 12 h prior to harvest. and subjected to IB at 48 h post infection (**A**) or at the indicated time points (**B**) with GAPDH as a loading control. Analysis of SAMHD1 promoter luciferase activity in 293T cells transfected with IRF3 and PA for 24 h. The results are expressed as the fold increase of luciferase activity in IRF3 and empty vector overexpression cells. The error bars represent data from three independent repeats (mean ± SD, *p < 0.05, **p < 0.01, ***p < 0.001, paired t test)

Since both the protein and mRNA of SAMHD1 are downregulated after H5N1infection, we speculate that H5N1 may downregulate SAMHD1 by affecting the amount of mRNA or by directly degrading SAMHD1 protein. To verify this conjecture, we first used inhibitors to check whether the H5N1 virus could degrade SAMHD1 at the protein level. Proteasome and lysosome pathways are the two main methods of protein degradation in cells. Since both the protein and mRNA of SAMHD1 may be downregulated by H5N1, we first treated A549 cells with proteasome inhibitor MG132, lysosome inhibitor chloroquine (CQ), or 3-methyladenine (3-MA) to elucidate whether H5N1 downregulates SAMHD1 expression through the above protein degradation pathways. The inhibitors were added to infected or uninfected A549 cells, and SAMHD1 and NP protein levels were detected 24 h after infection. The results showed that H5N1 did not downregulate SAMHD1 expression through the above protein degradation pathway (Fig. 6A, B). The protein level of SAMHD1 can be regulated not only at the translation stage but also by promoter activity at the transcriptional stage. The viral protein PA has been reported to inhibit the activation of IRF3 [60], and IRF3 is one of the factors that regulate the activity of the SAMHD1 transcriptional promoter [61]. Therefore, we hypothesized that the viral protein PA can regulate the expression of SAMHD1 by affecting the activity of IRF3. For this purpose, SAMHD1 promoter luciferase activity was assessed in 293T cells transfected with SAMHD1-Luc, IRF3 and PA plasmids. The luciferase activity in cells was detected 24 h later. The results showed that transfection of PA protein significantly downregulated the promoter activity of SAMHD1 (Fig. 6C), which means that H5N1 protein PA affects the expression level of SAMHD1 protein by regulating its transcription process.

### SAMHD1 can restrict H5N1 replication

**Fig. 7:**
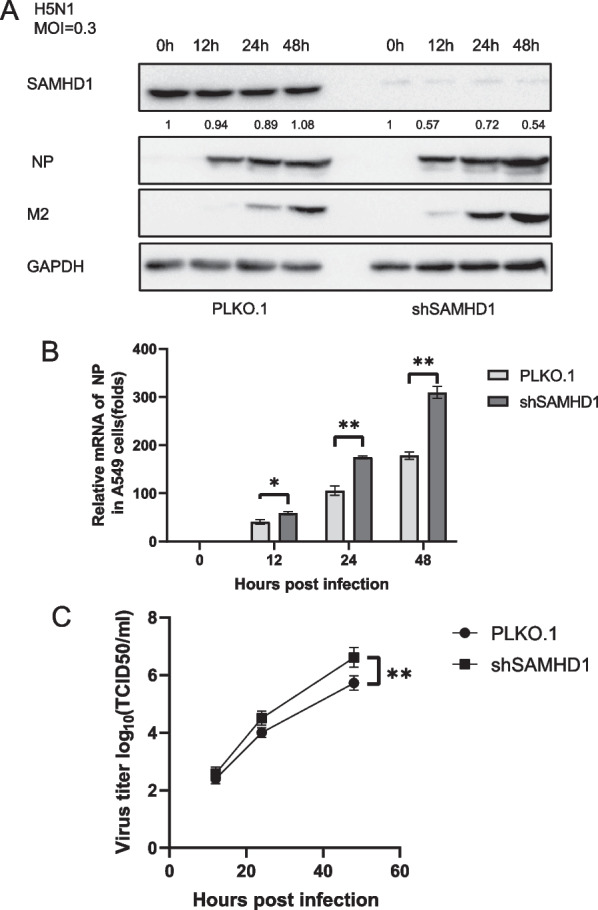
H5N1 replication was enhanced in SAMHD1 knockdown cells. Stable A549 cell lines generated by transfecting control vector (PLKO.1) and sh-SAMHD1 constructs were infected with H5N1 at an MOI of 0.3 (**A**). The cells and culture supernatants were harvested at the indicated time points. Immunoblotting analysis of NP, M2 and SAMHD1-Flag in cells was performed with GAPDH as a loading control. Viral RNA in cell lysates was detected by RT‒qPCR and normalized to GAPDH, and the viral RNA level in A549 cells at 0 h post infection was set as 1 (**B**). Viral titers of H5N1 in the supernatants were measured by the cytopathic effect method (**C**). The results are representative of those from three independent repeats (mean ± SD, ns stands for no significance, * *p* < 0.05, ** *p* < 0.01, paired t test)

**Fig. 8:**
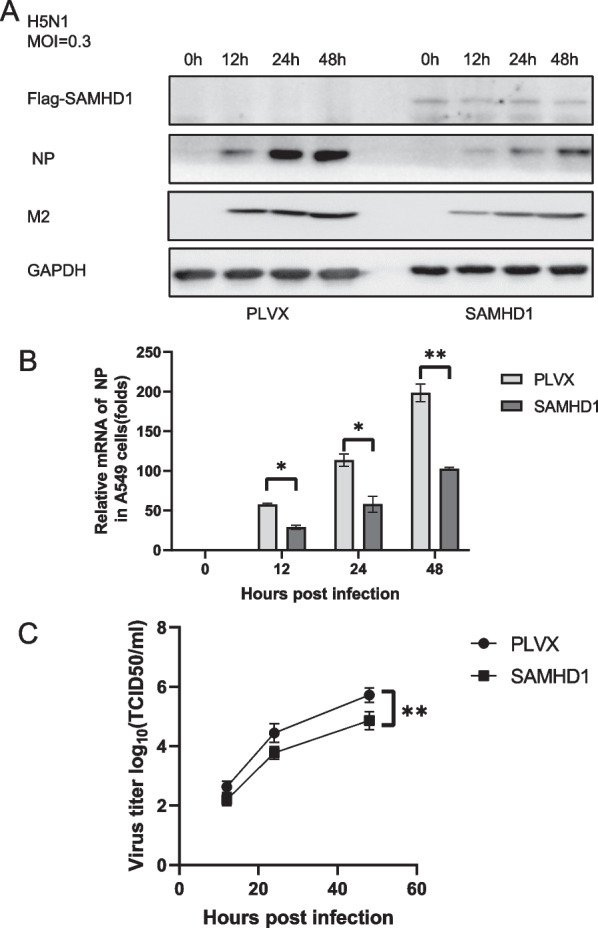
H5N1 replication was weakened in SAMHD1 overexpressed cells. Stable A549 cell lines generated by transfecting control vector (PLVX) and PLVX-SAMHD1-FLAG constructs were infected with H5N1 at an MOI of 0.3 (**A**), and the cells and culture supernatants were harvested at the indicated time points. Immunoblotting analysis of NP, M2 and SAMHD1 in cells was performed with GAPDH as a loading control. Viral RNA in cell lysates was detected by RT‒qPCR and normalized to GAPDH, and the viral RNA level in A549 cells at 0 h post infection was set as 1 (**B**). Viral titers of H5N1 in the supernatants were measured by the cytopathic effect method (**C**). The results are representative of those from three independent repeats (mean ± SD, ns stands for no significance, * *p* < 0.05, ** *p* < 0.01, paired t test).

Since H5N1 downregulates the expression of SAMHD1 by affecting SAMHD1 mRNA transcriptional promoter activity, we speculated that SAMHD1 may have the ability to restrict the replication of H5N1, and then the virus downregulates the expression of SAMHD1 to resist the host’s restriction in response. To verify the hypothesis that SAMHD1 can restrict against H5N1 replication, SAMHD1 overexpression and knockdown A549 cell lines were constructed in this study and were infected with H5N1 at 0.3 MOI. Viral protein NP and SAMHD1 expression levels were detected at different time points. We observed that viral protein NP in SAMHD1 knockdown cells was significantly higher than that in control cells (Fig. 7A). The mRNA level of NP protein exhibited similarity to that of the protein and was significantly enhanced in SAMHD1 knockdown cells compared to control cells (Fig. 7B). By detecting the virus titer in the cell culture supernatant, we found that the virus titer in the supernatant of cells with SAMHD1 knocked down was significantly higher than that of control cells (Fig. 7C). Unlike SAMHD1 knocked down cells, in overexpressing SAMHD1 cell lines, viral protein NP and M2 is lower than that in control cells (Fig. 8A). The level of virus mRNA and the amount of virus in the culture supernatant in the overexpressing SAMHD1 cell lines were significantly reduced compared to the control cells (Fig. 8B, C), which indicated that H5N1 replication can be significantly inhibited by SAMHD1. The results show that SAMHD1 can inhibit the replication of H5N1 and may be a restriction factor of H5N1.

### SAMHD1 restriction was associated with phosphorylation at threonine 592

**Fig. 9:**
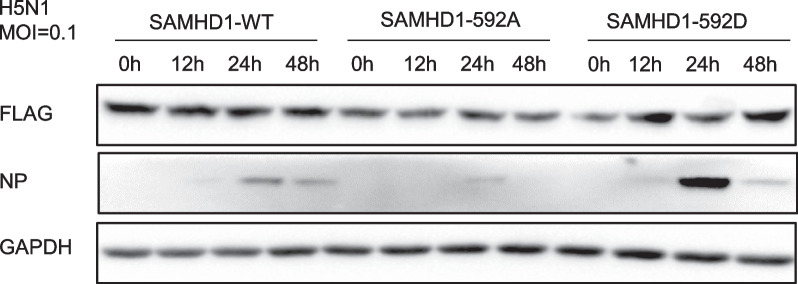
SAMHD1 restriction of H5N1 replication was associated with phosphorylation at T592. Stable A549 cell lines generated by transfecting PLVX-SAMHD1-FLAG, PLVX-SAMHD1-T592A-FLAG or PLVX-SAMHD1-T592D-FLAG plasmids were infected with H5N1 at an MOI of 0.1, and the cells were harvested at the indicated time points. Immunoblotting analysis of NP and SAMHD1-Flag in cells was performed with GAPDH as a loading control

SAMHD1 is a host restriction factor, and its function in restricting other reported viruses is related to phosphorylation at threonine 592 (T592). In HIV, the antiviral activity of SAMHD1 is negatively regulated by phosphorylation at the T592 residue [58]. For EV71, SAMHD1 phosphorylated at T592 loses the ability to restrict EV71 replication, which is similar to the restriction in HIV [59]. To investigate whether the anti-H5N1 capability of SAMHD1 is associated with the phosphorylation of tyrosine at position 592, we engineered A549 cells to stably express dephosphorylation mutants of SAMHD1 (T592A) and phosphomimetic mutants of SAMHD1 (T592D). Following this, we infected these cells with H5N1 and collected cell samples at various time points to assess virus replication (Fig. 9). To achieve a significant viral suppression effect, the infection MOI was set to 0.1. The results showed that dephosphorylation mutant T592A SAMHD1-expressing cells had stronger inhibition of H5N1 than the wild type, while the phosphorylated mutant T592D had weaker inhibition of H5N1 (Fig. 9). This means that SAMHD1's ability to inhibit H5N1 is related to phosphorylation at T592, which negatively regulates H5N1, similar to HIV-1 and EV71, which suggests that SAMHD1 may have the same mechanism to suppress the above three viruses.

## Conclusion and discussion

The viral protein HA first binds to sialic acid (SA) on the surface of the respiratory epithelial cell membrane. AIVs prefer to bind to the SA linked to galactose in the form of alpha-2,3, while human IAVs (H1, H2, H3) prefer to bind to SA linked to galactose in the form of alpha-2,6 [62]. After IAV enters the susceptible cell, the conformation of the virus M2 protein changes, the cation channel opens, and the pH in the virus particle decreases, causing the viral ribonucleoprotein complexes (vRNP) to detach from M1 matrix protein (M1) and release into the cytoplasm [63, 64]. In vRNP, nucleoprotein (NP) binds to viral RNA, and the viral RNA polymerase complex binds to the end of viral RNA. Proteins in the vRNP complex are localized to the nucleus through Importinα and Importinβ mediated classical nuclear input pathways for replication and transcription [65, 66]. Viral mRNA is then exported to the cytoplasm, along with the expression of viral proteins. M1 proteins can bind to vRNA and NP proteins, facilitating the assembly of vRNP complexes [67]. The NS2 protein can form a chain structure with the M1 and vRNP complex to guide vRNP out of the nucleus. The exported vRNP complex will first accumulate in the microtubule tissue center of the cytoplasm and be transported to the plasma membrane through the circulating endosome. With the help of HA, NA, M2 and other proteins, infectious virions will be formed on the membrane and released out of the cell through budding [68].

Many host restrictions have shown anti-influenza functions in several ways, such as virus entry (IFITM2, IFITM3) [69], genomic replication (OAS/RNaseL) [70], protein translation (PKR) [71, 72] and virus egress (BST-2) [73]. Our group has previously found that TIP60 can inhibit IAV replication through the TBK1-IRF3 pathway [74]. In this study, we found that SAMHD1 can restrict the replication of H5N1 and that H5N1 can downregulate the expression of SAMHD1, thus antagonizing the antiviral effect of host cells. First, we detected that the expression level of SAMHD1 increased slightly and then decreased in A549 cells infected with H5N1 and H1N1 Fig. 1 (A, B), Fig. 4, Fig. 5 , suggesting that SAMHD1 may be involved in the antiviral mechanism of host cells, and its downregulation may be due to the influence of IAVs infection. We then determined that SAMHD1 inhibits H5N1 replication and is a host restriction factor for IAV H5N1 by using SAMHD1 knockdown and overexpressed A549 cell lines (Figs. 7A, 8A). In these results, we also found an inconsistency between the protein level and mRNA level of SAMHD1. After H5N1 infection, the protein and mRNA levels of SAMDH1 first increased and then decreased. However, at 48 h, the protein level was significantly lower than that at 0 h, while the mRNA level at 48 h was not, which is inconsistent with the WB results. This may be because the expression level of SAMD1 protein is not only related to the mRNA level but also subject to posttranscriptional regulation, such as through the UTR. In 293T cells, the intracellular SMHD1 mRNA level at 48 h was significantly lower than that in uninfected cells. The reason for the different changes in SAMHD1 in the two types of cells is not clear, and it may be due to SAMHD in 293T cells mainly regulating the amount of SAMHD1 protein through changes in mRNA levels. Thus far, we have established the antiviral function of SAMHD1, but its specific antiviral mechanism is not clear. SAMHD1 may exert antiviral function directly or through the downstream pathway of SAMHD1, which may be one of the next research directions.

**Fig. 10:**
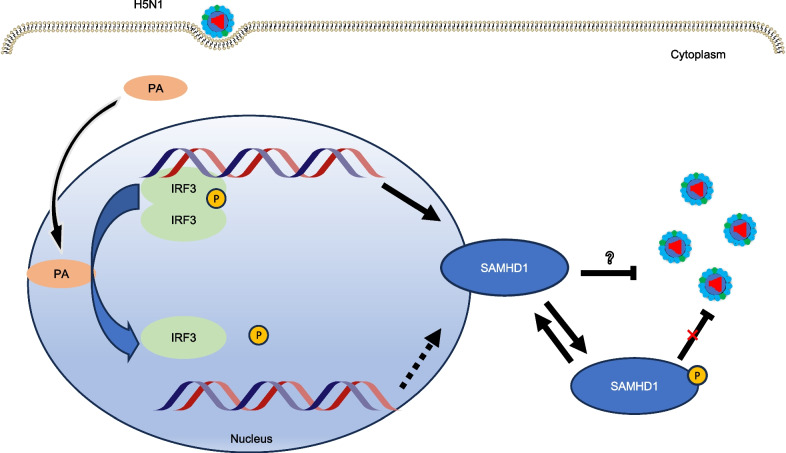
Proposed inhibition mechanism of host SAMHD1 on H5N1 replication. PA dephosphorylates IRF3, resulting in decreased expression of SAMHD1, which is resistant to the antiviral effect of SAMHD1. The antiviral effect of SAMHD1 was negatively correlated with its phosphorylation. PA had no significant effect on the phosphorylation of SAMHD1

Since PA protein can bind to IRF3 to inhibit its activity and IRF3 can upregulate SAMHD1 expression by increasing SAMHD1 promoter activity, we hypothesize that H5N1 virus PA protein downregulates SAMHD1 expression through a similar mechanism. This was demonstrated by the results that PA can downregulate the SAMHD1 transcription levels by downregulating IRF3 activity (Fig. 6C). SAMHD1 is phosphorylated at T592 by CDK/cyclin D/E in the G1-like phase [75]. We previously found that the T592D mutant, the SAMHD1 phosphorylation mimic, lost its EV71 restriction activity [57]. Therefore, we examined the effect of SAMHD1 phosphorylation at T592 on its antiviral ability and found that the ability of SAMHD1 to restrict IAV H5N1 is negatively regulated by T592 phosphorylation (Fig. 10), which may be one of the sites that regulate the antiviral ability of SAMHD1. SAMHD1 can inhibit the IFN-I pathway [35] and may play a role when IFN-I expression is too high. Therefore, the downregulation of SAMHD1 expression by PA can not only counter the antiviral effect of SAMHD1 but also affect the downregulation of IFN-I by SAMHD1 and its downstream pathway, which is believed to play an antiviral function and is produced after virus infection. To explore treatment against IAV, the strategy by which IAV regulates IFN deserves further study.

In conclusion, following viral infection, the virion constituents, particularly viral nucleic acids, are sensed by pathogen recognition receptors (PRRs). These receptors use adaptor proteins to stimulate a signal transduction pathway that sequentially triggers the next molecule downstream in the pathway ultimately culminating in the phosphorylation activation of IRF3 and the expression of IFN and SAMHD1 genes. The PA protein will be able to dephosphorylate IRF3, thereby inactivating it and thus downregulating the subsequent expression of SAMHD1 (Fig. 10).

## Data Availability

The datasets used or analysed during the current study are available from the corresponding author on reasonable request.

## Abbreviations

IAV: Influenza A virus
AIV: Avian influenza virus
MOI: Multiplicity of infection
WB: Western Blot
HPAI: Highly pathogenic avian influenza
PRRs: Pattern recognition receptors
IFN-I: Type I interferon
NS1: Nonstructural protein 1
ISGs: Interferon stimulator genes
AGS: Aicardi-Goutières syndrome
LTR: Long terminal repeats
NLS: Nuclear localization sequence
EV71: Enterovirus 71
CQ: Chloroquine
3-MA: 3-Methyladenine
SA: Sialic acid
vRNP: Viral ribonucleoprotein complexes
